# Genomic and Transcriptomic Analysis Reveal Multiple Strategies for the Cadmium Tolerance in *Vibrio parahaemolyticus* N10-18 Isolated from Aquatic Animal *Ostrea gigas Thunberg*

**DOI:** 10.3390/foods11233777

**Published:** 2022-11-23

**Authors:** Pan Yu, Lianzhi Yang, Juanjuan Wang, Chenli Su, Si Qin, Chaoxi Zeng, Lanming Chen

**Affiliations:** 1 Key Laboratory of Quality and Safety Risk Assessment for Aquatic Products on Storage and Preservation (Shanghai), Ministry of Agriculture and Rural Affairs of the People’s Republic of China, College of Food Science and Technology, Shanghai Ocean University, Shanghai 201306, China; 2 Lab of Food Function and Nutrigenomics, College of Food Science and Technology, Hunan Agricultural University, Changsha 410128, China

**Keywords:** *Vibrio parahaemolyticus*, cadmium tolerance, genome, transcriptome, antibiotic resistance, aquatic product

## Abstract

The waterborne *Vibrio parahaemolyticus* can cause acute gastroenteritis, wound infection, and septicemia in humans. Pollution of heavy metals in aquatic environments is proposed to link high incidence of the multidrug-resistant (MDR) pathogen. Nevertheless, the genome evolution and heavy metal tolerance mechanism of *V. parahaemolyticus* in aquatic animals remain to be largely unveiled. Here, we overcome the limitation by characterizing an MDR *V. parahaemolyticus* N10-18 isolate with high cadmium (Cd) tolerance using genomic and transcriptomic techniques. The draft genome sequence (4,910,080 bp) of *V. parahaemolyticus* N10-18 recovered from *Ostrea gigas Thunberg* was determined, and 722 of 4653 predicted genes had unknown function. Comparative genomic analysis revealed mobile genetic elements (*n* = 11) and heavy metal and antibiotic-resistance genes (*n* = 38 and 7). The bacterium significantly changed cell membrane structure to resist the Cd^2+^ (50 μg/mL) stress (*p* < 0.05). Comparative transcriptomic analysis revealed seven significantly altered metabolic pathways elicited by the stress. The zinc/Cd/mercury/lead transportation and efflux and the zinc ATP-binding cassette (ABC) transportation were greatly enhanced; metal and iron ABC transportation and thiamine metabolism were also up-regulated; conversely, propanoate metabolism and ribose and maltose ABC transportation were inhibited (*p* < 0.05). The results of this study demonstrate multiple strategies for the Cd tolerance in *V. parahaemolyticus*.

## 1. Introduction

*Vibrio parahaemolyticus* is a Gram-negative bacterium that thrives in marine, riverine, and aquaculture environments worldwide [1,2]. The bacterium can cause acute gastroenteritis, wound infection, and septicemia in humans [2]. *V. parahaemolyticus* was first identified as a foodborne pathogen in Japan in the 1950s [3]. Since then, pathogenic *V. parahaemolyticus* has been reported in Asian countries and subsequently in Africa, America, and Europe, arguing a pandemic of *V. parahaemolyticus* worldwide [4]. It was estimated that *V. parahaemolyticus* is responsible for roughly 35,000 human infection cases each year in the United States [5]. The bacterium has been identified as the leading cause of the foodborne diarrhea disease in China since the 1990s [6]. The crucial virulence determinants in pathogenic *V. parahaemolyticus* are thermostable-direct hemolysin (TDH) and TDH-related hemolysin (TRH) [7].

*V. parahaemolyticus* is frequently isolated from aquatic products worldwide, including crustaceans, fish, and shellfish [1,8,9,10]. For example, recently, Li et al. analyzed food samples (*n* = 905) collected from 15 provinces in China and found that 14.17% of fish (*n* = 204), 15.34% of shrimp (*n* = 365), and 3.67% of ready-to-eat food (*n* = 300) samples were detected positive for potential *V. parahaemolyticus* contamination [8]. Antibiotics effectively control infectious diseases caused by pathogenic microorganisms in aquaculture [11]. Nevertheless, during the past few decades, the inappropriate application of antibiotics in medical treatment and aquaculture has resulted in the emergence and spread of multidrug resistant (MDR) pathogenic bacteria, which pose serious threats to therapeutic options for human diseases [9]. On the other hand, rapid industrialization and modernization may lead to the increased heavy metal pollution in the aquatic environment, such as cadmium (Cd), chromium (Cr), copper (Cu), lead (Pb), mercury (Hg), nickel (Ni), and zinc (Zn). Heavy metal residues are detected in various aquatic environments and aquatic products [12,13]. The high bioaccumulation of heavy metals through the food chain poses grave risks to human health. For example, Cd is classified into Group 1 as carcinogenic to humans by the International Agency for Research on Cancer (IARC) [14]. Moreover, heavy metals have been proposed to enhance selection for antibiotic resistance in the environment and vice versa [15]. Recently, Fang et al. isolated *V. parahaemolyticus* strains (*n* = 112) from Pacific mackerel (*Pneumatophorus japonicus*) samples (*n* = 360) collected from different markets in Zhejiang Province, China. They found that most strains showed resistance to the antibiotics ampicillin (AMP) (*n* = 108) and streptomycin (STR) (*n* = 101), as well as the heavy metals Cd^2+^ (*n* = 88) and Pb^2+^ (*n* = 58) [16].

The co-selection is highly favored when diverse resistance genes are located on the same mobile genetic elements (MGEs) [17,18]. Genetic diversity of MGEs and genome plasticity in *V. parahaemolyticus* may affect the survival of the pathogen in the environment [19]. With the increased breakthrough of sequencing technology [20], approximately 1740 *V. parahaemolyticus* isolates have been sequenced, among which complete genomes of 64 *V. parahaemolyticus* isolates are available in the GenBank database (https://www.ncbi.nlm.nih.gov/, accession date: 29 January 2022). Recently, draft genome sequences of six *V. parahaemolyticus* isolates recovered from six species of aquatic animals, *Paphia undulate*, *Perna viridis*, *Mactra veneriformis*, *Aristichthys nobilis*, *Carassius auratu*, and *Litopenaeus vannamei*, were determined by our research group; a complete genome sequence of *V. parahaemolyticus* CHN25 was also obtained [21,22].

In our previous studies, a number of *V. parahaemolyticus* strains were isolated and characterized from various species of aquatic animals [23]. Of these, *V. parahaemolyticus* N10-18 isolate showed MDR and high tolerance to Cd^2+^ and Zn^2+^. Therefore, we asked what the genome features of this bacterium could be and what the molecular mechanism underlying the resistance phenotype could be. Thus, the major objectives of this study were (1) to determine the draft genome sequence of *V. parahaemolyticus* N10-18 isolate recovered from the aquatic animal *Ostrea gigas Thunberg* using Illumina Hiseq × 10 sequencing technique; (2) to identify MGEs and virulence- and resistance-related genes in the *V. parahaemolyticus* N10-18 genome; (3) to examine the survival of *V. parahaemolyticus* N10-18 at different concentrations of Cd^2+^ stress; (4) to decipher the possible molecular mechanism of the Cd^2+^ tolerance in *V. parahaemolyticus* N10-18 by comparative transcriptomics analysis. The results of this study will enrich genome data of *V. parahaemolyticus* and facilitate the risk control of the leading seafood-borne pathogen in edible aquatic animals.

## 2. Materials and Methods

### 2.1. V. parahaemolyticus Strain and Cultural Conditions

*V. parahaemolyticus* N10-18 strain was isolated from *O. gigas Thunberg* and identified in our previous research [23] (Table S1). The bacterium was routinely incubated in Tryptic Soy Broth (TSB) medium (3% NaCl, pH 8.5, Beijing Land Bridge Technology Co., Ltd., Beijing, China) at 37 °C with shaking at 180 rpm. The bacterial growth was examined as described in our previous study [24].

### 2.2. Genomic DNA Preparation, Sequencing, Assembly, and Annotation

*V. parahaemolyticus* N10-18 incubated in the TSB medium to the middle-logarithmic growth phase (mid-LGP) was harvested by centrifugation at 8000× *g* for 1 min. Genomic DNA of the bacterial cell pellet was extracted using the MiniBEST DNA extraction kit (Japan TaKaRa BIO, Dalian Company, Dalian, China) according to the manufacture’s instructions. DNA concentrations and purity (A_260_/A_280_) of the extracted samples were examined as described previously [25]. High-quality DNA samples were subjected to genome DNA sequencing, which was conducted by Shanghai Majorbio Bio-pharm Technology Co., Ltd., Shanghai, China, using the Illumina HiSeq × 10 sequencing platform (Illumina, Santiago, CA, USA). The average length of sequencing reads was 150 bp. Raw sequencing reads were analyzed using the FastQC software (https://www.bioinformatics.babraham.ac.uk/projects//fastqc/, accessed on 30 January 2022) [26] with the parameters described in our previous research [21]. High-quality reads were assembled using the SOAPdenovo (version 2.04) software [21]. *V. parahaemolyticus* RIMD2210633 (GenBank accession numbers for chromosomes 1 and 2: NC_004603.1 and NC_004605.1) was used for the reference genome. Coding sequences (CDSs), rRNA, and tRNA genes were predicted using the software Glimmer (version 3.02) [27], Barrnap tool (https://github.com/tseemann/barrnap, accessed on 30 January 2022), and tRNAscan-SE (version 2.0) [28] with 80% identity and 90% coverage at E ≤ 1 × 10^−5^, respectively.

Functional assignments of the predicted CDSs were inferred [29]. If the CDS did not match the clusters of orthologous groups (COG) function, it was assigned as unknown protein. The programs were run with default parameters.

### 2.3. Comparative Genome Analysis

The average nucleotide identity (ANI) value was calculated using the JspeciesWS software (http://jspecies.ribohost.com/jspeciesws/, accessed on 28 August 2022). MGEs were described in our previous studies [21,24,30], and genome islands (GIs), prophages, integrons (INs), and insertion sequences (ISs) were predicted using the software IslandViewer (version 1.2) [31], Phage_Finder [32], Integron_Finder (version 2.0) [33], and ISEScan (version 1.7.2.1) [34], respectively. The virulence factor database (https://www.mgc.ac.cn/VFs, accessed on 28 August 2022), heavy metal resistance gene database BacMet (http://bacmet.biomedicine.gu.se/, accessed on 28 August 2022), and antibiotic resistance gene database (http://ardb.cbcb.umd.edu/, accessed on 28 August 2022) were employed to analyze virulence-, heavy metal-, and antibiotic-resistance-related genes, respectively.

Gene loci of somatic (O) antigens and capsular polysaccharide (K) antigens are located between *dgkA* and *gmhD* genes and between *gmhD* and *rjg* genes in *V. parahaemolyticus* genomes, respectively [35,36]. Of these, the *wvaG*, *wvaR*, *VP0208*, *orf16*, *wvcA*, *wvcJ*, *wvcN*, *wvdG*, *wvaH*, *wvcP/orf9*, *wvdB*, and *wvcP* genes are responsible for the O1 to O12 serotype antigens, respectively [35], while the *VP0214* to *VP0238* genes are for the K serotype antigens, respectively [36]. Serotype sequences of 12 O antigens and 43 K antigens were collected from *V. parahaemolyticus* ATCC17802, RIMD221063, and 20-082a3 strains that are available in current literature [35,36,37,38,39,40]. The strains with untypeable (UT) antigenic markers by the Basic Local Alignment Search Tool (BLAST) (https://www.ncbi.nlm.nih.gov/BLAST, accessed on 30 August 2022) analysis were designated as OUT (O untypeable) or KUT (K untypeable).

Based on seven conserved core genes *dnaE*, *gyrB*, *recA*, *dtdS*, *pntA*, *pyrC*, and *tnaA* in *V. parahaemolyticus* [41], the multi-locus sequence typing (MLST) analysis was performed using the MLST software (version 2.19.0, http://github.com/tseemann/mlst, accessed on 15 February 2022).

### 2.4. Phylogenetic Tree Analysis

Complete gene sequences of 64 *V. parahaemolyticus* strains were downloaded from the GenBank database (Table S2). Amino acid data sets of single-copy orthologs in *V. parahaemolyticus* genomes were analyzed using the software OrthoFinder (version 2.2.6) [42]. The FastTree (version 2.1.11) software was used to build a phylogenetic tree using the method and parameters described in our recent research [21].

### 2.5. Determination of Minimum Inhibitory Concentrations (MICs) of Antibiotics and Heavy Metals

The MICs of antibiotics and heavy metals against *V. parahaemolyticus* N10-18 were measured using the broth dilution testing (microdilution) according to the guidelines of the Clinical and Laboratory Standards Institute (CLSI, M2-A9, 2006), including the CdCl_2_, ZnCl_2,_ AMP, kanamycin (KAN), and STR (Sinopharm Chemical Reagent Co., Ltd., Shanghai, China). *Escherichia coli* K-12 was used as a quality control strain in the tests [23].

### 2.6. Stress Conditions

The fresh cell culture of *V. parahaemolyticus* N10-18 was individually inoculated into the TSB medium supplemented with different concentrations (0, 50, 100, 200, and 400 μg/mL) of CdCl_2_ and then incubated at 37 °C for 48 h. Bacterial growth curves were measured [24]. Bacterial survival rates were calculated using the standard colony counting method [43].

### 2.7. Cell Membrane Permeability, Fluidity, and Surface Hydrophobicity Assays

*V. parahaemolyticus* N10-18 was incubated in the TSB medium (3% NaCl, pH 8.5) to the mid-LGP at 37 °C. A final concentration of CdCl_2_ (50 μg/mL) was added and then incubated at 37 °C for 2 h. The outer cell membrane permeability was examined using the method described by Harman et al. [44]. The N-phenyl-1-naphthylamine (NPN) was purchased from the Shanghai Labtop Bio-Technology Co., Ltd., Shanghai, China. The inner membrane permeability was examined using the method described by Ibrahim et al. [45]. The O-nitrophenyl-β-D galactopyranoside (ONPG) was purchased from Beijing Solarbio Science & Technology Co., Ltd., Beijing, China.

The membrane fluidity assay was performed using the method described by Voss and Montville [46]. The cell surface hydrophobicity assay was performed using the method described by Yan et al. [47]. The n-hexadecane was purchased from China National Pharmaceutical Group Corporation Co., Ltd., Shanghai, China.

### 2.8. Scanning Electron Microscope (SEM) Assay

The SEM assay was performed according to the method described previously [48]. Briefly, a final concentration (50 μg/mL) of CdCl_2_ was added into *V. parahaemolyticus* N10-18 culture grown in the TSB medium (pH 8.5, 3% NaCl) at mid-LGP and then continuously incubated at 37 °C for 2 h. An amount of 1.5 mL of the cell suspension was collected, washed, dehydrated, dried, and gold-covered by cathodic spraying and observed using the thermal field emission SEM (Hitachi, SU5000, Tokyo, Japan) with accelerating voltages of 5–10 kV [48].

### 2.9. Illumina RNA Sequencing and Analysis

*V. parahaemolyticus* N10-18 was incubated in the TSB medium (pH 8.5, 3% NaCl) to the mid-LGP at 37 °C. A final concentration of CdCl_2_ (50 μg/mL) was added and then incubated at 37 °C for 2 h. Controls were cultures also exposed to no cadmium for the same time period and collected as treatments. The bacterial cells were collected by centrifugation and subjected for the Illumina RNA sequencing. The RNA extraction and quality control, sequencing library construction, and Illumina sequencing were conducted by Shanghai Majorbio Bio-pharm Technology Co., Ltd., Shanghai, China, using Illumina HiSeq 2500 platform (Illumina, Santiago, CA, USA). Three replicates were conducted for each sample.

Expression of each gene was calculated, and differentially expressed genes (DEGs) were defined and used for gene set enrichment analysis (GSEA) as described previously [25]. Representative DEGs were examined using real-time reverse-transcription PCR (RT-qPCR) assay [25,48].

### 2.10. Statistical Analysis

The data were analyzed using the SPSS software (version 22, IBM, Armonk, NY, USA). All tests in this study were conducted in triplicate.

## 3. Results

### 3.1. Genotype and Phenotype of V. parahaemolyticus N10-18

*V. parahaemolyticus* N10-18 isolate was recovered from *O. gigas Thunberg* [23]. The bacterium tested negative for the toxic *tdh* and *trh* genes but positive for the species-specific gene *tlh* [49]. The results also showed that *V. parahaemolyticus* N10-18 was tolerant to the heavy metals Cd^2+^ and Zn^2+^, as well as the antimicrobial agents AMP, KAN, and STR (Table S1).

### 3.2. Genome Features of V. parahaemolyticus N10-18

**Figure 1:**
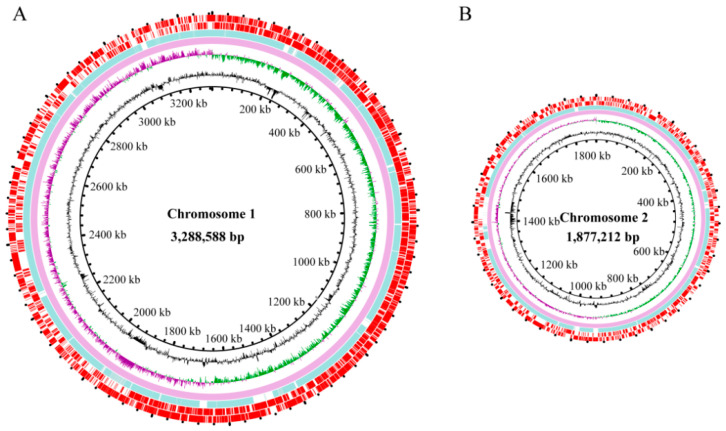
Genome circle maps of *V. parahaemolyticus* N10-18. (**A**,**B**) represent the larger and smaller chromosomes of *V. parahaemolyticus* N10-18, respectively. Circles from the inside to outside: GC contents (outward part means higher than average, inward part means lower than average); GC skew (purple value is greater than zero, green value is less than zero); the reference genome of *V. parahaemolyticus* RIMD2210633 (GenBank accession numbers: NC_004603.1 and NC_004605.1) and *V. parahaemolyticus* N10-18 genome (GenBank accession no. JALGSE000000000), respectively; and CDSs on the positive and negative chains, respectively.

The ANI value of the *V. parahaemolyticus* N10-18 genome was determined, which was higher (98.22%) than the threshold (94–96%) for species determination [50]. The draft genome sequence of *V. parahaemolyticus* N10-18 was determined using the Illumina Hiseq × 10 sequencing technique (Figure 1), and approximately 438,181 clean single reads were obtained. The assembly generated 70 scaffolds with a sequencing depth (on average) of 319.27-fold. *V. parahaemolyticus* N10-18 showed a clean single peak in the frequency of observed unique 17-mers within the sequencing data and varied as a typical Poisson distribution, suggesting less repetitive DNA in the *V. parahaemolyticus* N10-18 genome (Figure S1). The obtained *V. parahaemolyticus* N10-18 genome sequence has been deposited in the GenBank database under the assigned accession number JALGSE000000000.

The obtained genome size of *V. parahaemolyticus* N10-18 was 4,910,080 bp with 45.46% of the GC content. A total of 4653 genes were predicted, among which approximately 4565 coded for proteins. Remarkably, approximately 722 proteins-coding genes had unknown function, while 3843 were classified into 21 functional catalogues against the COG database (Table 1).

The *V. parahaemolyticus* N10-18 genome contained transposase genes (*n* = 10) and MGEs, including GIs (*n* = 2), INs (*n* = 8), and ISs (*n* = 1), suggesting possible horizontal gene transfer (HGT) mediated by the MGEs during the *V. parahaemolyticus* N10-18 genome evolution. The identified MGEs were absent from the ends of the scaffolds (Table S3), which indicated that the draft genome contained all such elements.

### 3.3. Serotype and ST of V. parahaemolyticus N10-18

The BLAST analysis of the antigen gene loci revealed that the *V. parahaemolyticus* N10-18 genome contained the O antigen loci *orf16*/*wvdB* and specific loci *wzc* for K4 polymorphic sites [51], indicating that the serotype of *V. parahaemolyticus* N10-18 was O4/O11:K4. Additionally, the ST by the MLST analysis showed that the bacterium belonged to the ST-499.

### 3.4. Phylogenetic Relatedness of V. parahaemolyticus N10-18

**Figure 2:**
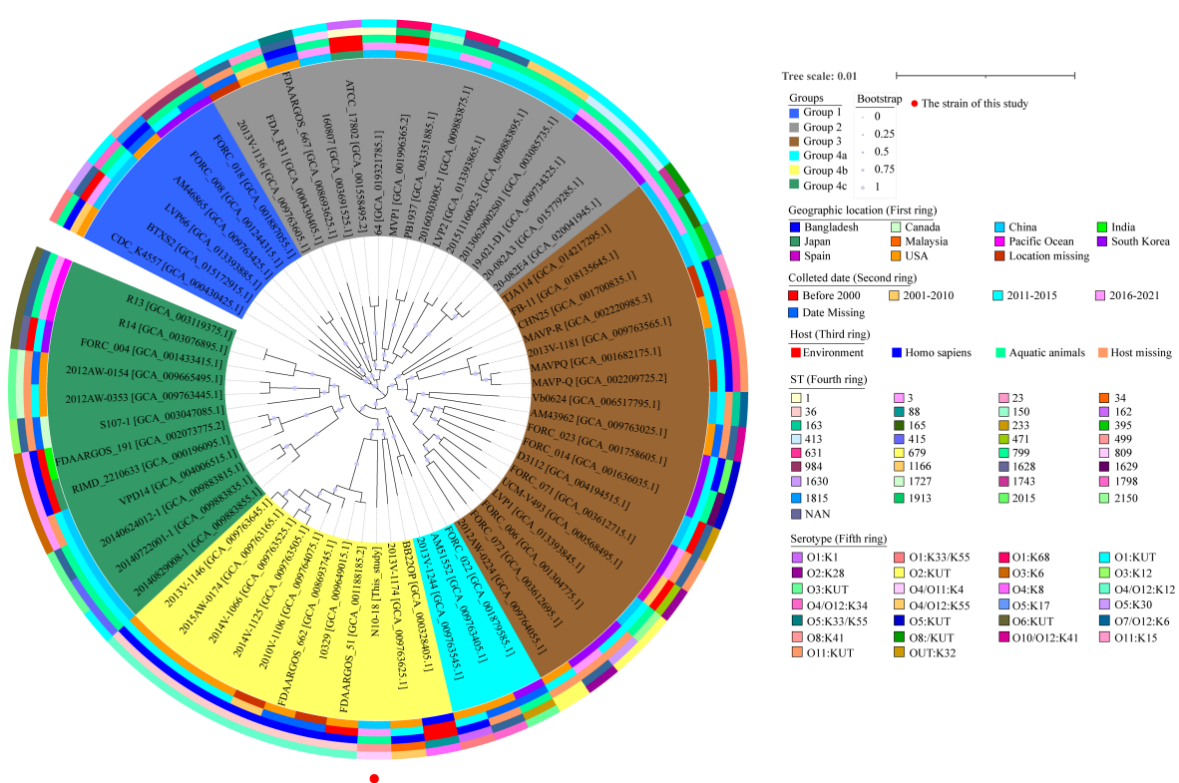
A phylogenetic tree construed on the basis of genome-wide homologous single-copy genes in 65 *V. parahaemolyticus strains*. The isolation time and location, serotypes, STs, and host information of these strains were integrated into the tree.

Approximately 1485 homologous single-copy amino acid sequences were identified from 64 *V. parahaemolyticus* genomes available in the GenBank database and the *V. parahaemolyticus* N10-18 genome determined in this study. A phylogenetic tree was construed (Figure 2), in which 20 *V. parahaemolyticus* strains were recovered from homo sapiens, 7 from the environment, 26 from aquatic animals (Penaeus, crayfish, fine spotted flounder, marinated crab, oyster, seabass, shrimps, and toothfish), and 12 from an unknown source (Table S2). This analysis revealed four distinct groups, designated as Groups 1 to 4. Group 4 was further classified into three subgroups (Groups 4a, 4b, and 4c) (Figure 2).

Although *V. parahaemolyticus* N10-18 (O4/O11:K4; ST-499; GenBank accession no. JALGSE000000000) was classified into Group 4b, the bacterium fell into a single sub-branch and was found to be phylogenetically distant from the other *V. parahaemolyticus* strains originating in aquatic animals. Moreover, *V. parahaemolyticus* N10-18 showed the closest evolutionary distance with the *V. parahaemolyticus* strains FDAARGOS_51, 10,329, FDAARGOS_662, 2010V-1106, 2014V-1125, 2014V-1066, 2015AW-0174, and 2013V-1146 with the GenBank assembly accession nos. GCA_001188185.2, GCA_009649015.1, GCA_008693745.1, GCA_009764075.1, GCA_009763505.1, GCA_009763525.1, GCA_009763165.1, and GCA_009763645.1, respectively. These strains were isolated from Homo sapiens between 1998 and 2015 in the USA (except *V. parahaemolyticus* 2010V-1106 and 10,329 strains with unknown isolation location) and belonged to O4/O12:K12, and ST-36 (Figure 2). These results demonstrated the unique genome trait of *V. parahaemolyticus* N10-18 with the resistance phenotype and provided additional evidence for the genome variation of *V. parahaemolyticus* in aquatic animals.

### 3.5. MGEs in the V. parahaemolyticus N10-18 Genome

#### 3.5.1. GIs

**Figure 3:**
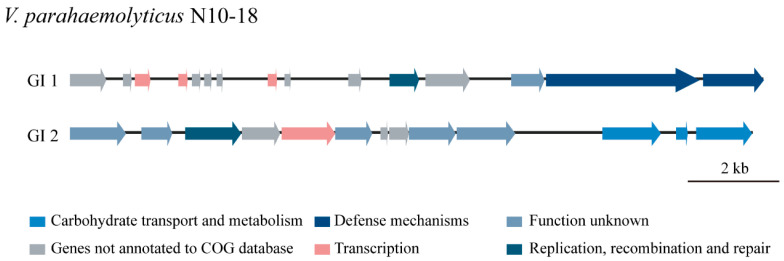
The gene organization of the GIs identified in the *V. parahaemolyticus* N10-18 genome. Different colors refer to COG classification to mark gene function, and genes with unknown function are displayed in grey color.

GIs play a critical role in *V. parahaemolyticus* genome evolution by the acquisition of novel biological traits through HGT [52]. In this study, two GIs (GI 1 to GI 2) were identified in the *V. parahaemolyticus* N10-18 genome (Figure 3). GI 1 (15,200 bp) contained 15 genes, wherein six had known functions, encoding a serine/threonine protein phosphatase (*Vp_N10_18_3246*), a transcriptional regulator (*Vp_N10_18_3248*), cold-shock proteins (*Vp_N10_18_3249*, *Vp_N10_18_3253*), a resolvase (*Vp_N10_18_3256*), and a deoxyribonuclease HsdR (*Vp_N10_18_3259*). The other nine genes coded for unknown proteins. GI 2 (14,954 bp) had 13 genes, wherein seven had known functions, encoding a short-chain dehydrogenase (*Vp_N10_18_3808*), an integrase (*Vp_N10_18_3810*), a transcriptional regulator (*Vp_N10_18_3812*), a P-loop ATPase (*Vp_N10_18_3817*), a dehydrogenase (*Vp_N10_18_3818*), a phosphotransferase system (PTS) system cellobiose-specific IIB component (*Vp_N10_18_3819*), and a PTS sugar transporter (*Vp_N10_18_3820*). The other six genes coded for unknown proteins.

#### 3.5.2. INs

**Figure 4:**
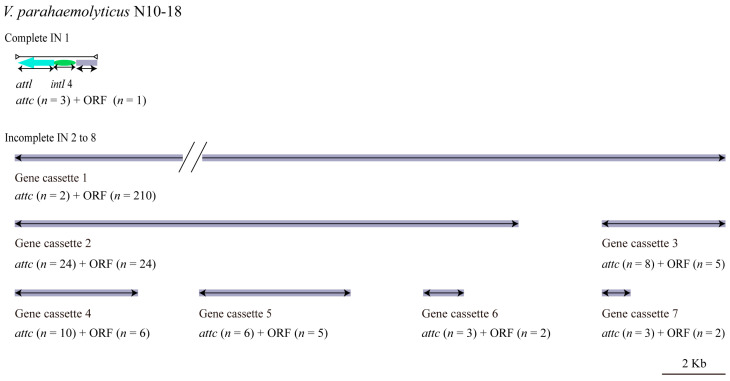
The structure diagram of the INs identified in the *V. parahaemolyticus* N10-18 genome. The complete IN and incomplete gene cassettes are shown with the predicted *attc/attl* sites and ORFs.

Mobile INs are prevalent in human-dominated ecosystems with prolonged exposure to selective agents such as detergents, antibiotics, and heavy metals [53]. INs are generally classified according to integrase genes *intI* 1, *intI* 2, *intI* 3, and *intI* 4 into type I, type II, type III, and super integron, respectively [54]. In this study, eight INs (IN 1 to IN 8) were identified in the *V. parahaemolyticus* N10-18 genome, which ranged from 910 bp to 227,599 bp and carried 2 to 210 genes. Of these, there was one complete IN (IN 1) and seven gene cassettes (IN 2 to IN 8) (Figure 4). Typically, gene cassettes consist of a promoterless open reading frame (*orf*) and a recombination site (*attC*) necessary for integration. They can exist free as circular molecules or mobilized in INs [55].

In this study, the complete IN 1 (2566 bp) contained a hypothetical protein-encoding gene (*Vp_N10_18_2516*) and an integrase gene *IntI* 4 (*Vp_N10_18_2515*). The latter showed sequence identity (99.38%) with the super IN *IntI* 4 (NR reference sequence: AHI99301.1) [56], which indicated that IN 1 was a super IN in *V. parahaemolyticus* N10-18.

Among the seven incomplete INs, the largest, IN 2 (227,599 bp), contained 210 genes, of which 54 genes coded for hypothetical proteins. IN 3 (15,877 bp) carried 24 genes encoding 15 hypothetical proteins and 9 proteins with known functions including an adenylate kinase and related kinase (*Vp_N10_18_4543*), a GCN5-related N-acetyltransferase (GNAT) (*Vp_N10_18_4551*), a histone acetyltransferase (*Vp_N10_18_4552*), plasmid stabilization proteins (*Vp_N10_18_4545* and *Vp_N10_18_4553*), prevent-host-death family proteins (*Vp_N10_18_4544* and *Vp_N10_18_4554*), a putative membrane protein (*Vp_N10_18_4549*), and a site-specific DNA-methyltransferase (*Vp_N10_18_4538*). Additionally, IN 4 (3898 bp) carried five genes encoding four hypothetical proteins and a vco30 (*Vp_N10_18_4598*); IN 5 (3866 bp) encoded five hypothetical proteins and an acetyltransferase (*Vp_N10_18_4601*); IN 6 (4779 bp) encoded four hypothetical proteins and a methyltransferase (*Vp_N10_18_4620*); IN 7 (1300) encoded two hypothetical proteins; and IN 8 (910 bp) encoded a hypothetical protein and a plasmid stabilization protein ParE (*Vp_N10_18_4638*) (Figure 4).

Virulence-related genes were also detected in the INs in *V. parahaemolyticus* N10-18, such as the GNAT (*Vp_N10_18_4551*, IN 3), prevent-host-death family proteins (*Vp_N10-18_4544* and *Vp_N10-18_4554*, IN 3), and plasmid stabilization proteins (*Vp_N10_18_4545* and *Vp_N10_18_4553*, IN 3; *Vp_N10-18_4638*, IN 8).

#### 3.5.3. ISs

A single short IS can transfer one or more resistance-related genes in Gram-negative bacteria and affect bacterial resistance phenotype [21,22]. In this study, only one IS110 (1327 bp) was identified in the *V. parahaemolyticus* N10-18 genome, encoding a IS110 family transposase (Table S3).

### 3.6. Putative Virulence-Associated Genes

We identified approximately 45 virulence-associated genes in the *V. parahaemolyticus* N10-18 genome by the BLAST analysis (Table S4). Of these, 36 genes encoded type III secretion system 1 (T3SS1)-related proteins, including VecA, YscO, VcrDGHRV, VopBDNQRS, and VscCDFGHIJKLNPQRSTUVWXY. T3SS1 is an essential virulence determinant for *V. parahaemolyticus* survival in the environment [57,58]. Interestingly, the *exsACD* gene cascade was also present in the *V. parahaemolyticus* N10-18 genome. T3SS1 expression is regulated by this cascade, in which the master transcription factor ExsA positively regulates T3SS1 expression, whereas ExsD negatively regulates its expression [59]. The other virulence-associated genes functioning in bacterial adhesion or epithelial cell invasion also existed in the *V. parahaemolyticus* N10-18 genome, e.g., *ilpA* [60], *MAM7* (multivalent adhesion molecule 7) [61], *gmhA* [62], *gmd* [63], and *kdsA* [64] (Table S4).

### 3.7. Heavy Metal and Antibiotic Resistance-Associated Genes

Approximately 38 heavy metal tolerance-associated genes were identified in the *V. parahaemolyticus* N10-18 genome by the BLAST analysis (Table 2). For example, the *cadC* gene and *dsbABC* gene cluster, which are responsible for the bacterial tolerance to Cd, Zn, and Pb, as well as Cd, Zn, Hg, and Cu, respectively [65], were present in the *V. parahaemolyticus* N10-18 genome. Moreover, the *zntAR, znuABC, zur,* and *smtA* genes for the Zn and Hg tolerance [65,66,67] were also identified (Table 2). These results were consistent with the heavy metal tolerance phenotype of *V. parahaemolyticus* N10-18.

Antimicrobial resistance-associated genes (*n* = 7) also existed in the *V. parahaemolyticus* N10-18 genome (Table 2), such as an elongation factor Tu (*tuf*) [68], a cAMP-activated global transcriptional regulator CRP (*crp*) [69], a DNA-directed RNA polymerase subunit beta (*rpoB*) [70], and a hexose-6-phosphate (*uhpT*) [71], *tet34* and *tet35* [72], and β-lactamase (*bla_CARB-21_*), consistent with the MDR phenotype of *V. parahaemolyticus* N10-18.

### 3.8. Survival of V. parahaemolyticus N10-18 under the Cd^2+^ (50 μg/mL) Stress

Based on the above results, MIC values of the heavy metals and antibiotics against *V. parahaemolyticus* N10-18 were determined (Table S1). Remarkably, the observed MICs of Cd^2+^ and Zn^2+^ were 400 μg/mL and 1600 μg/mL, respectively. Given that Zn^2+^ is essential for the growth and development of aquatic animals and often used as feed additives, the survival of *V. parahaemolyticus* N10-18 to resist the high level of Cd^2+^ was further investigated in this study.

**Figure 5:**
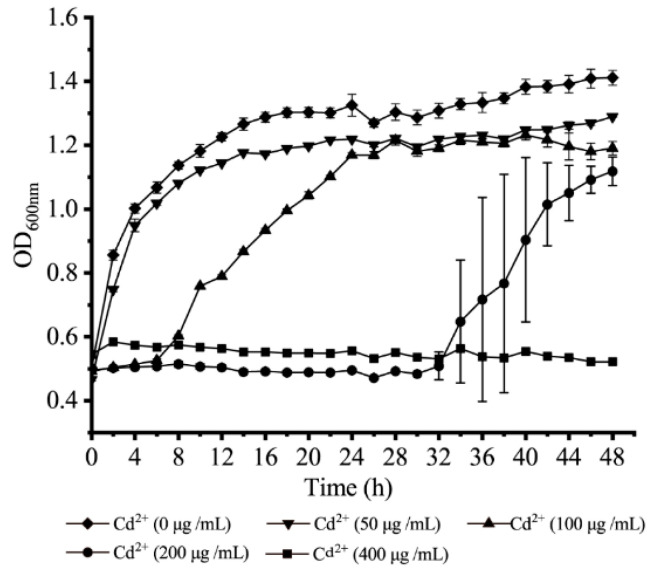
The survival of *V. parahaemolyticus* N10-18 under different concentrations of heavy metal Cd^2+^. Three replicates were assessed at each concentration.

Growth curves of *V. parahaemolyticus* N10-18 at different concentrations of CdCl_2_ were determined in the TSB medium at 37 °C. As shown in Figure 5, at the concentration of 400 μg/mL of Cd^2+^, the growth of *V. parahaemolyticus* N10-18 was fully inhibited. At 200 μg/mL and 100 μg/mL of Cd^2+^, the bacterial growth was retarded, showing a longer lag phase of 32 h and 6 h, respectively. Moreover, the bacterial biomass reached the maximum with OD_600 nm_ values of 1.178 and 1.216 at 48 h and 28 h, respectively. At 50 μg/mL of Cd^2+^, only a slight decrease in growth was observed, when compared with the control (0 μg/mL of Cd^2+^). Under this treatment, the observed fatality rate of *V. parahaemolyticus* N10-18 was 10.73%.

### 3.9. Changes in Cell Membrane Permeability and Fluidity and Cell Surface Hydrophobicity of V. parahaemolyticus N10-18 under the Cd^2+^ (50 μg/mL) Stress

**Figure 6:**
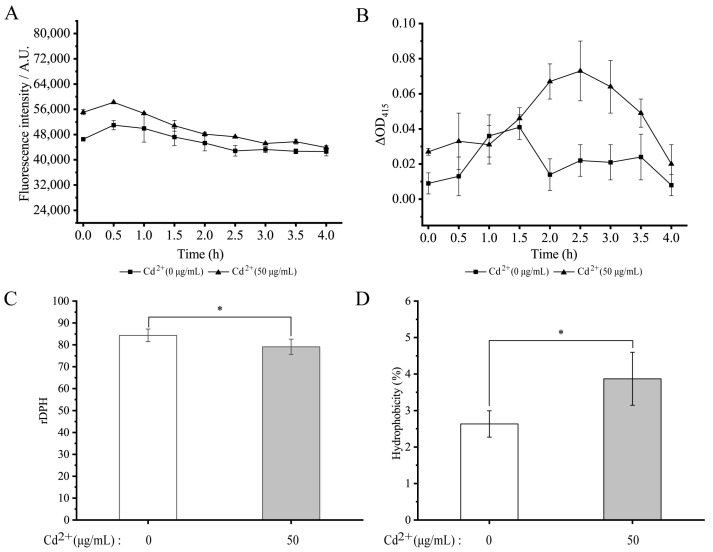
The changes in cell membrane permeability and fluidity and cell surface hydrophobicity of *V. parahaemolyticus* N10-18 under the Cd^2+^ (50 μg/mL) stress. (**A**–**D**) The outer and inner membrane permeability, membrane fluidity, and cell surface hydrophobicity, respectively. * *p* < 0.05.

Bacterial cell membrane permeability and fluidity and cell surface hydrophobicity are key parameters of the cell membrane responding to environmental challenges [80,81]. Cd is a heavy metal whose cations often cause toxicity to both eukaryotic and prokaryotic cells even at low concentrations [82]. In this study, the outer cell membrane permeability was examined using the NPN probe. As shown in Figure 6A, the probe fluorescence intensity showed an overall downward trend after treatment with 50 μg/mL of Cd^2+^ for 4 h. Additionally, we used the ONPG as a probe to examine the inner cell membrane permeability, and no significant difference in the inner membrane permeability was also observed after the Cd^2+^ stress for 1.5 h compared with the control group (*p* > 0.05). However, the extended treatment time (2.0 to 4.0 h) increased the bacterial inner membrane permeability by 2.04- to 4.96-fold (*p* < 0.05) (Figure 6B).

Cytoplasmic membrane fluidity also influences the ability of most compounds (nutrients and antibiotics) and ions to cross the bacterial cytoplasmic membrane by diffusion and active transport [80]. As shown in Figure 6C, cell membrane fluidity of *V. parahaemolyticus* N10-18 was significantly decreased by 1.07-fold after being treated with 50 μg/mL of Cd^2+^ for 2 h, compared with the control group. Cell surface hydrophobicity is crucial in bacterial adhesion to abiotic and biological surfaces [83]. As shown in Figure 6D, cell surface hydrophobicity of *V. parahaemolyticus* N10-18 was significantly increased by 1.47-fold after being treated with 50 μg/mL of Cd^2+^ for 2 h (*p* < 0.05).

**Figure 7:**
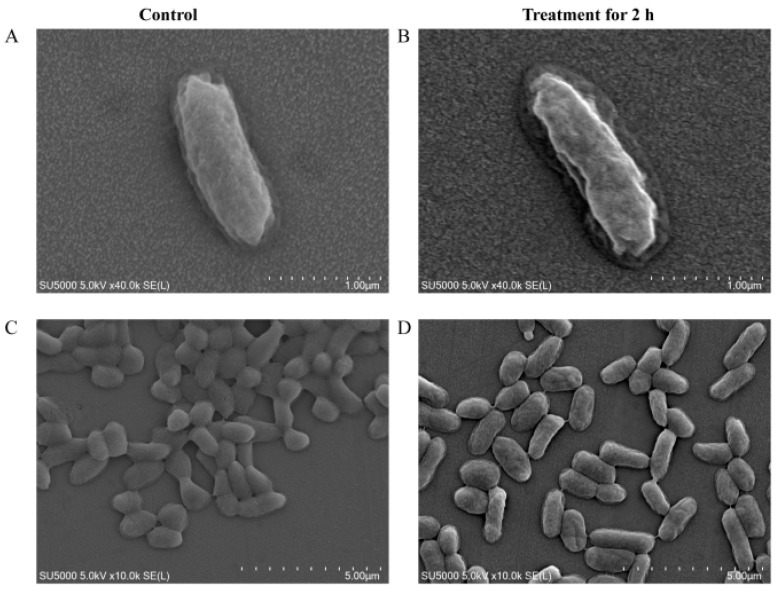
The SEM observation of cell surface structure of *V. parahaemolyticus* N10-18 under Cd^2+^ (50 μg/mL) stress. (**A**,**C**) The control groups with 0 μg/mL of Cd^2+^ (observed by ×40.0 k, and ×10.0 k). (**B**,**D**) The treatment groups with 50 μg/mL of Cd^2+^ (observed by ×40.0 k, and ×10.0 k).

Cell structure changes of *V. parahaemolyticus* N10-18 under the Cd^2+^ (50 μg/mL) stress were also observed by the SEM analysis. As shown in Figure 7, the treatment with Cd^2+^ (50 μg/mL) for 2 h resulted in the cell surface shrinking of certain *V. parahaemolyticus* N10-18 cells compared to the control group.

### 3.10. The Major Changed Metabolic Pathways Medicated by the Cd^2+^ (50 μg/mL) Stress in V. parahaemolyticus N10-18

Based on the obtained results, *V. parahaemolyticus* N10-18 grown at the mid-LGP in the TSB medium at 37 °C was treated with the Cd^2+^ (50 μg/mL) for 2 h, and gene expression changes at the global genome level of *V. parahaemolyticus* N10-18 induced by the Cd^2+^ stress were determined using the Illumina HiSeq 2500 sequencing technology.

**Figure 8:**
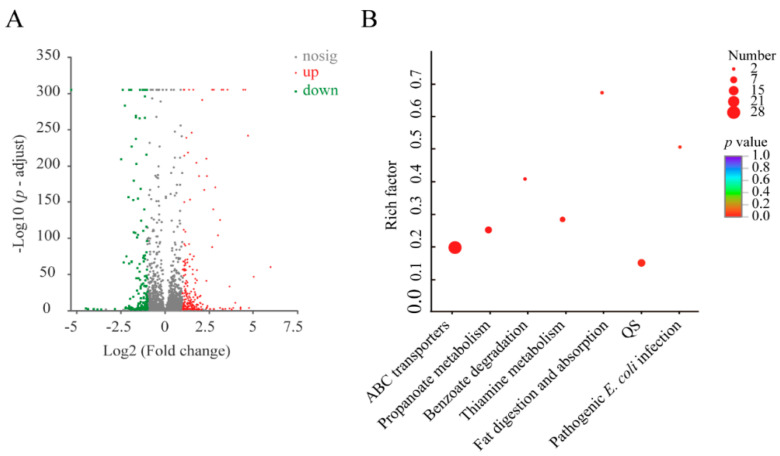
The major changed metabolic pathways in *V. parahaemolyticus* N10-18 under the Cd^2+^ (50 μg/mL) stress. (**A**) The volcano plot of differential gene expression. (**B**) The significantly altered metabolic pathways in the bacterium. The pathway of ‘pathogenic *E. coli* infection’ was labeled according to the catalog in the Kyoto Encyclopedia of Genes and Genomes (KEGG) database.

Approximately 8.3% (377 of 4565 genes) of the bacterial genes were differentially expressed under the treatment, when compared to the control group. Of these, 217 DEGs showed higher transcriptional levels (fold change ≥ 2.0), whereas 160 were significantly down-regulated (fold change ≤ 0.5) (Figure 8A). Approximately seven significantly altered metabolic pathways were identified in *V. parahaemolyticus* N10-18, including the ATP-binding cassette (ABC) transporters, propanoate metabolism, benzoate degradation, thiamine metabolism, fat digestion and absorption, quorum sensing (QS), and pathogenic *E. coli* infection (Figure 8B, Table 3).

Remarkably, the expression of approximately 28 DEGs of the ABC transporters was significantly changed at the transcription level (0.061- to 11.609-fold) (*p* < 0.05). Of these, the DEGs encoding the maltose and ribose transporters and some amino acid transporters were significantly inhibited (0.061- to 0.500-fold). For example, the *malEK* gene cluster (*Vp_N10_18_1557*, *Vp_N10_18_1556*), which encoded a maltose ABC transporter substrate-binding protein MalE and a maltose/maltodextrin import ATP-binding protein MalK, respectively, was significantly down-regulated. The *rbsBCD* gene cluster (*Vp_N10_18_3026*, *Vp_N10_18_3025*, and *Vp_N10_18_3023*), which encoded a ribose ABC transporter substrate-binding protein RbsB, a ribose ABC transporter permease, and a D-ribose pyranase, respectively, was significantly down-regulated as well (*p* < 0.05). Additionally, the expression of the *livH* gene (*Vp_N10_18_2959*), which encoded a branched-chain amino acid ABC transporter permease, was remarkably down-regulated (0.061-fold) at the transcriptional level.

In the propanoate metabolism, expression of approximately seven DEGs was also significantly inhibited (0.069- to 0.438-fold) (*p* < 0.05), e.g., the *prpCEF*, and *acnD* genes (*Vp_N10_18_0015*, *Vp_N10_18_0011*, *Vp_N10_18_0012*, and *Vp_N10_18_0013*) involved in the 2-methylcitric acid cycle (2-MCC). The 2-MCC in the propionate catabolic pathway is used to oxidize the Cα methylene of propionate to a keto group yielding pyruvate [84]. These results indicated that *V. parahaemolyticus* N10-18 greatly reduced the branched-chain amino acid transportation, inhibited the maltose and ribose transportation, and inactivated to utilize the propionic acid as a carbon source under the Cd^2+^ (50 μg/mL) stress.

Conversely, the DEGs encoding the Zn/Cd/Hg/Pb-transporting ATPase (*zntA*, *Vp_N10_18_0526*) and heavy metal efflux resistance-nodulation-cell division (RND) transporter of the CusA/CzcA family (*cusA*, *Vp_N10_18_0582*) were greatly up-regulated by 23.639- and 8.649-fold, respectively (*p* < 0.05). Moreover, the *znuABC* gene cluster (*Vp_N10_18_4099, Vp_N10_18_1679*, *Vp_N10_18_1681*, *Vp_N10_18_4101,* and *Vp_N10_18_1680*) involved in Zn uptake was highly expressed at the transcriptional level (2.594- to 11.609-fold) (*p* < 0.05). Of these, the *znuA* gene (*Vp_N10_18_1679*) was remarkably up-regulated by 11.609-fold. Cd is chemically similar to Zn, both of which belong to the IIB transition elements. It is probably common in microbial species that Cd^2+^ is imported via the Zn channels [67]. Likewise, with expression of three DEGs for the iron (III) transportation, which encoded a Fe^3+^-hydroxamate ABC transporter permease FhuB (*fhuB*, *Vp_N10_18_1520)*, an iron (III) ABC transporter ATP-binding protein (*Vp_N10_18_1522)*, and an iron ABC transporter substrate-binding protein (*afuA*, *Vp_N10_18_1887*), all were significantly increased (2.243- to 3.891-fold) (*p* < 0.05). Additionally, the *artIMP* gene cluster (*Vp_N10_18_0733*, *Vp_N10_18_0735*, and *Vp_N10_18_0732*) for the arginine ABC transportation was also significantly up-regulated (3.101- to 4.015-fold) (*p* < 0.05), which encoded an arginine ABC transporter substrate-binding protein, an arginine transporter permease subunit ArtM, and an arginine ABC transporter ATP-binding protein ArtP in the arginine transportation. Arginine is a structurally stabilizing factor that contains side chains to form salt bridges and hydrogen bonds [85].

In the QS, the DEGs encoding a sugar ABC transporter ATP-binding protein (*Vp_N10_18_1219*), a polyamine ABC transporter substrate-binding protein (*Vp_N10_18_0181*), and ABC transporter permeases (*Vp_N10_18_1876* and *Vp_N10_18_2783*) were also significantly up-regulated by 2.140- to 9.727-fold (*p* < 0.05).

In the thiamine metabolism, approximately five DEGs were also up-regulated at the transcriptional level (2.116- to 2.740-fold) (*p* < 0.05), which encoded a phosphomethylpyrimidine synthase (*thic*, *Vp_N10_18_4412*), a hydroxymethylpyrimidine kinase/phosphomethylpyrimidine kinase (*thiD*, *Vp_N10_18_1089*), thiamine phosphate synthases (*thiE*, *Vp_N10_18_1096* and *Vp_N10_18_4413*), and a thiaminase II (*tenA*, *Vp_N10_18_1094*). Thiamine is a precursor of thiamine pyrophosphate (TPP), an essential coenzyme in the central metabolism. Bacterial thiamine biosynthesis and salvage genes are controlled at the RNA level by TPP-responsive riboswitches that include the ABC family transporter ThiXYZ [86]. In this study, the *thiXYZ* genes (*Vp_N10_18_1091*, *Vp_N10_18_1092,* and *Vp_N10_18_1090*) were also up-regulated by 2.400- to 3.567-fold. These results suggested that the Cd^2+^ (50 μg/mL) stress enhanced the thiamine metabolism in *V. parahaemolyticus* N10-18 to increase the biosynthesis of TPP.

Interestingly, the DEGs encoding the T3SS needle filament protein VscF (*Vp_N10_18_0060*) and glyceraldehyde-3-phosphate dehydrogenase (*gapA*, *Vp_N10_18_3876*) in pathogenic *E. coli* infection were significantly up-regulated by 5.836- and 2.086-fold, respectively (*p* < 0.05). The VscF in T3SS1 helps to translocate VPA0226 that can be secreted into the host cell cytoplasm via T3SS1 in *V. parahaemolyticus* [58]. The *gapA* gene was only expressed under certain stress conditions, and overproduction of GapA led to increased resistance to H_2_O_2_ in *Lactococcus lactis* MG1363 [87]. Additionally, expression of representative DEGs was examined by the RT-PCR assay, and the resulting data were generally consistent with the transcriptomic analysis (Tables S6 and S7).

Taken together, under the Cd^2+^ (50 μg/mL) stress, *V. parahaemolyticus* N10-18 employed multiple strategies for efficient transportation and exocytosis of Cd^2+^ to alleviate its cytotoxicity: (1) greatly enhanced the Zn/Cd/Hg/Pb-transportation and efflux; (2) up-regulated metal and iron ABC transportation; (3) enhanced the biosynthesis of TPP in the thiamine metabolism; (4) up-regulated the expression of stress-related proteins, such as GapA, and structurally stabilizing factor arginine; (5) conversely, greatly reduced the branched-chain amino acid transportation; (6) inhibited the maltose and ribose ABC transportation; and (7) down-regulated the propanoate metabolism, in order to survive in the adverse niche.

## 4. Discussion

The pollution of heavy metals in aquatic environments has led to heavy metal residues in aquatic products, which poses a huge hidden danger in human health [88,89]. Cd is classified into Group 1 as carcinogenic to humans by the IARC [14]. This toxic element possibly results in short-term or long-term disorders in the body, such as degenerative bone disease, kidney dysfunction, lung injuries, disorders in Zn and Cu metabolism, and cancer [14]. The heavy metal pollution has also been supposed to link high incidence of the MDR *V. parahaemolyticus*, which is a challenging issue in the clinical treatment. This study was the first to characterize the MDR *V. parahaemolyticus* N10-18 with high tolerance to Cd^2+^ and Zn^2+^. The observed MIC values of Cd^2+^, Zn^2+^, AMP, KAN, and STR against *V. parahaemolyticus* N10-18 were 400 μg/mL, 1600 μg/mL, 50,000 μg/mL, 128 μg/mL, and 128 μg/mL, respectively, which suggested possible antibiotic and heavy metal pollution in the aquaculture environment of *O. gigas Thunberg*, consistent with previous reports [8,15,21,23].

In this study, the draft genome sequence (4,910,080 bp) of *V. parahaemolyticus* N10-18 was determined using the Illumina Hiseq × 10 sequencing technique. Some repeats were located at the end of scaffolds (*n* = 23, <1.1 Kb) (Table S5), indicating that the genome assembly was incomplete and contained some gaps, due to limitations of the second-generation Illumina short-read sequencing technique. Approximately 4653 genes were predicted, of which 722 encoded unknown proteins. In our previous study, we found a number of unknown-function genes in *V. parahaemolyticus* isolates from the six species of aquatic animals (*P. undulate*, *P.a viridis*, *M.veneriformis*, *A. nobilis*, *C. auratu*, and *L. vannamei*) [21]. These results demonstrated genome variation and plasticity of *V. parahaemolyticus* in aquatic animals.

Intercellular transmissibility of MGEs may have constituted important driving forces in *V. parahaemolyticus* genome evolution and formation of ecotypes and speciation [90]. In this study, we identified 11 MGEs in the *V. parahaemolyticus* N10-18 genome. It cannot exclude that additional MGEs may be located in the gaps between scaffolds [21]. In this study, we found two GIs that contained 28 genes in the *V. parahaemolyticus* N10-18 genome, which facilitated the bacterium to better fit into the niche. For example, GI 1 contained the genes encoding cold-shock proteins (CSPs) (*Vp_N10_18_3249*, *Vp_N10_18_3253*). The CSPs served as global gene expression regulators to respond to different stress conditions [22].

INs allow bacteria to capture, stockpile, express, and exchange genes embedded within gene cassettes [91]. In this study, one super IN and seven incomplete INs were identified in the *V. parahaemolyticus* N10-18 genome. Although approximately 33.20% of the INs-carrying genes (*n* = 85) encoded unknown proteins, the identified INs endowed the bacterium with diverse environmental adaptability. For instance, several gene cassettes were found to carry virulence-related genes, such as the GNAT super family proteins, prevent-host-death family proteins, and plasmid stabilization proteins. Of these, the GNAT (*Vp_N10_18_4551*) belongs to type II toxin of toxin–antitoxin systems. The GNAT toxin blocks protein translation by acetylating the amino group of charged tRNAs, thus preventing tRNA from participating in peptidyl ribosomal transferase [92].

Some *V. parahaemolyticus* isolates lacking the *tdh* and/or *trh* genes are also highly cytotoxic to human gastrointestinal cells, which indicates that other virulence factors exist. In this study, we found potential virulence-related genes (*n* = 45) in the *V. parahaemolyticus* N10-18 genome, e.g., *ilpA*, *MAM7*, *exsACD*, *gmhA*, *gmd*, *kdsA*, and T3SS1-related genes, which are involved in bacterial adhesion or epithelial cell invasion. For example, the *ilpA* gene encodes an immunogenic lipoprotein A in *Vibrio vulnificus,* an adhesion molecule that can induce cytokine production in human immune cells [60]. MAM7 enables Gram-negative pathogens to establish high-affinity binding to host cells during the early stages of infection and is crucial for the delivery of virulence factors into hosts [61]. In this study, we identified 36 genes in T3SS1 in the *V. parahaemolyticus* N10-18 genome, which are important determinants of the pathogenicity of *V. parahaemolyticus*. Of these genes, *VscCD* genes not only activated bacterial resistance to acid stress, H_2_O_2_, and antibiotics but also enhanced the colonization ability and pathogenicity of *Vibrio harveyi* [93]. These results suggested a health risk in consuming *O. gigas Thunberg* contaminated by *V. parahaemolyticus* N10-18.

Bacterial MDR is regarded as an emerging pollutant in different food production avenues including aquaculture [94]. Resistance factors have been reported in pathogenic bacteria [68,69,70,71,72,95,96,97,98]. In this study, we identified seven antibiotic-resistance-related genes in the *V. parahaemolyticus* N10-18 genome, consistent with the observed MDR phenotype of the bacterium. Remarkably, 38 heavy metal tolerance-associated genes existed in *V. parahaemolyticus* N10-18. For instance, the gene (*Vp_N10_18_3808*, GI 2) encoding a short-chain dehydrogenase/reductase SDR family member was identified, which functions in the Cd^2+^ stress in *Pleurotus eryngii* [99]. Moreover, the *cadC*, *dsbABC*, *zntAR*, *znuABC*, *zur*, and *smtA* genes related to the Cd and Zn resistance were also present in the *V. parahaemolyticus* N10-18 genome, consistent with the high Cd and Zn tolerance phenotype of the bacterium. For instance, the *dsbABC* gene cluster involved in the degradation of pyrimidine ribonucleosides was found to be related to the resistance and absorbing of Cd in *Enterococcus faecalis* LZ-11, which was isolated from Lanzhou reach of the Yellow River in China [100]. The diversity of resistance genes, gene variance, and selective pressure from the environment may result in the difference between resistance phenotype and resistance genotype.

In this study, the constructed phylogenetic tree showed that the 65 *V. parahaemolyticus* genomes were clustered into four large clusters, among which *V. parahaemolyticus* N10-18 fell into a single sub-branch in Group 4b. The bacterium is located phylogenetically distant from the other *V. parahaemolyticus* strains originating in aquatic animals but showed the closest evolutionary distance with 8 *V. parahaemolyticus* strains isolated from Homo sapiens between 1998 and 2015 in the USA. Until 1996, *V. parahaemolyticus* infection cases were sporadic, occurred in certain countries, and could be related to diverse serovars [101]. Location and isolation time of *V. parahaemolyticus* strains were not associated with evolutionary taxa, suggesting that the widespread global trade in aquatic products over the past 30 years may have contributed to the cross-regional spread of the pathogen, leading to an increased risk of edible aquatic animals.

Based on the findings in this study, the molecular mechanism underlying the heavy metal Cd^2+^ tolerance of *V. parahaemolyticus* N10-18 was further explored. Under the Cd^2+^ (50 μg/mL) stress, the bacterium significantly changed cell membrane permeability and fluidity and cell surface hydrophobicity (*p* < 0.05). Cell osmotic changes have been disclosed as stressors that can affect biophysical properties and the composition of the membrane and consequently transport mechanisms (permeability) and cell shape and integrity [80]. In this study, after the Cd^2+^ (50 μg/mL) treatment, the cell surface of *V. parahaemolyticus* N10-18 was observed shrinking to a certain extent.

Comparative transcriptomic analysis revealed seven significantly altered metabolic pathways in *V. parahaemolyticus* N10-18 under the Cd^2+^ stress. Remarkably, the DEGs encoding the Zn/Cd/Hg/Pb-transporting ATPase (*zntA*), and heavy metal efflux RND transporter of the CusA/CzcA family (*cusA*) were greatly up-regulated by 23.639- and 8.649-fold, respectively (*p* < 0.05). The *zntA* gene was originally described as a Zn-transporting ATPase in *E. coli*, but it also confers resistance to Cd [66]. RND efflux pumps are essential for the expulsion of a plethora of potentially small lethal agents or compounds such as detergents, solvents, heavy metals, antibiotics, and toxic secondary metabolites [102]. The CusC(F)BA complex exports copper (I) and silver (I) and mediates resistance to these two metal ions in *E. coli* [102]. Interestingly, in this study, the ABC transporter encoded by the *znuABC* genes for high-affinity Zn^2+^ uptake [66,67] was also highly increased at the transcriptional level (2.594- to 11.609-fold) (*p* < 0.05). This was consistent with the high tolerance of *V. parahaemolyticus* N10-18 to Zn^2+^. Given that Cd is chemically similar to Zn, both of which belong to the IIB transition elements [67], our result provided evidence for the expulsion of Cd^2+^ via the Zn^2+^ channels in *V. parahaemolyticus* N10-18. On the other hand, the DEGs (*afuA* and *fhuB*) encoding the ion and metal transporters were also up-regulated (*p* < 0.05). For instance, the *fhuB* gene encoded a Fe^3+^-hydroxamate ABC transporter permease FhuB. Iron (III) hydroxamate transport across the cytoplasmic membrane is catalyzed by the very hydrophobic FhuB protein and the membrane-associated FhuC protein [103]. These results indicated highly enhanced expulsion of Cd^2+^ by *V. parahaemolyticus* N10-18 to alleviate its cytotoxicity.

In the QS, expression of five DEGs was also significantly up-regulated by 2.14- to 9.727-fold (*p* < 0.05). Additionally, the *pcaC* gene (*Vp_N10_18_2971*) encoding the carboxymuconolactone decarboxylase family protein in benzoate degradation and the *atoB* gene (*Vp_N10_18_2988*) encoding the thiolase family protein in the fat digestion and absorption were also significantly enhanced by 2.003- and 4.215-fold, respectively (*p* < 0.05). These results suggested possibly increased substance absorption for energy conservation and stringent response regulation in *V. parahaemolyticus* N10-18 under the Cd^2+^ (50 μg/mL) stress.

In contrast, the DEGs involved in the branched-chain amino acid transportation and maltose and ribose transportation were significantly repressed (0.061- to 0.500-fold) (*p* < 0.05). Meanwhile, the propanoate metabolism was also significantly inhibited (0.069- to 0.438-fold) (*p* < 0.05). These results suggested possible repressed energy consumption and nucleotide and ribosome biosynthesis under the Cd^2+^ adverse condition. It will be interesting to further investigate the DEGs using proteomic, cell, and animal mode techniques and methods in the future research.

## 5. Conclusions

This study was the first to characterize the MDR *V. parahaemolyticus* N10-18 with high tolerance to Cd^2+^ and Zn^2+^ (MIC_S_: 400 μg/mL and 1600 μg/mL) using genomic and transcriptomic techniques. The draft genome sequence (4,910,080 bp) of *V. parahaemolyticus* N10-18 was determined, and 722 of 4653 predicted genes had unknown function. Comparative genomic analyses revealed MGEs, including GIs (*n* = 2), INs (*n* = 8), and ISs (*n* = 1). Heavy metal and antibiotic-resistance genes (*n* = 38 and 7) and virulence-associated genes (*n* = 45) were also found in the *V. parahaemolyticus* N10-18 genome. The bacterial growth was slightly decreased under the 50 μg/mL of Cd^2+^. *V. parahaemolyticus* N10-18 significantly changed cell membrane permeability and fluidity and surface hydrophobicity under the Cd^2+^ (50 μg/mL) stress (*p* < 0.05). Meanwhile, comparative transcriptomic analysis revealed seven significantly altered metabolic pathways. Under the Cd^2+^ stress, *V. parahaemolyticus* N10-18 employed multiple strategies for efficient transportation and exocytosis of Cd^2+^ to alleviate its cytotoxicity, including greatly enhanced Zn/Cd/Hg/Pb transportation and efflux and significantly up-regulated metal and iron ABC transportation, thiamine metabolism, and stress-related protein expression (e.g., GapA and arginine); in contrast, it greatly reduced the branched-chain amino acid transportation and significantly inhibited the maltose and ribose ABC transportation and propanoate metabolism, in order to resist and survive in the adverse Cd^2+^ environment. The results also provided evidence for the expulsion of Cd^2+^ via the Zn^2+^ channels in *V. parahaemolyticus* N10-18. Overall, the results of this study enriched genome data of *V. parahaemolyticus* from aquatic animals and revealed multiple strategies for the cadmium tolerance in the leading seafood-borne pathogen worldwide.

## Figures and Tables

**Table 1:** General features of the *V. parahaemolyticus* N10-18 genome.

Genome Feature	*V. parahaemolyticus* N10-18
Genome size (bp)	4,910,080
G + C (%)	45.46
DNA Scaffold	70
Total predicted gene	4653
Protein-coding gene	4565
RNA gene	143
Genes assigned to COG	3843
Genes with unknown function	722
Transposase gene	10
GI	2
Prophage	0
IN	8
IS	1

**Table 2:** The heavy metal and antibiotic resistance-related genes identified in the *V. parahaemolyticus* N10-18 genome.

Heavy Metal and Antibiotic Agent	Resistance Gene	Reference
Heavy metal		
As	*arsCR*, *pstABCS*	[65]
Cu	*actP*, *copAB*, *corC*, *cueR*, *cusABRS*, *cutAC*	[65,73]
Cr	*nfsA*	[65,74]
Ni	*nirBD*	[65,75]
Zn	*zntAR*, *znuABC*, *zur*	[66,67]
Zn, Hg	*smtA*	[65]
Cr, Fe	*chrAR*	[65,76]
W, Mo	*modABC*	[65]
Cr, Te, Se	*recG, ruvB*	[65]
Cd, Zn, Pb	*cadC*	[65,77]
Cd, Zn, Hg, Cu	*dsbABC*	[65]
Antimicrobial agent		
Beta-lactamases	*bla_CARB-21_*	[78]
Elfamycin	*tuf*	[68]
Fluoroquinolone	*crp*	[69]
Fosfomycin	*UhpT*	[71]
Peptide, rifamycin	*rpoB*	[70]
Tetracycline	*Tet (34)*, *Tet (35)*	[72,79]

**Table 3:** The major altered metabolic pathways in *V. parahaemolyticus* N10-18 under the Cd^2+^ (50 μg/mL) stress.

Metabolic Pathway	Gene	Gene ID	Fold Change	Description
ABC transporters	*livH*	*Vp_N10_18_2959*	0.061	Branched-chain amino acid ABC transporter permease
	*znuB*	*Vp_N10_18_4101*	0.081	Metal ABC transporter permease
	*malE*	*Vp_N10_18_1557*	0.252	Maltose ABC transporter substrate-binding protein MalE
	*malK*	*Vp_N10_18_1556*	0.263	Maltose/maltodextrin import ATP-binding protein MalK
	*rbsB*	*Vp_N10_18_3026*	0.325	Ribose ABC transporter substrate-binding protein RbsB
	*aapP*	*Vp_N10_18_2527*	0.355	Arginine ABC transporter ATP-binding protein
	*rbsD*	*Vp_N10_18_3023*	0.378	D-ribose pyranase
	*rbsC*	*Vp_N10_18_3025*	0.428	Ribose ABC transporter permease
	*aapJ*	*Vp_N10_18_2530*	0.441	Amino acid ABC transporter substrate-binding protein
	*oppF*	*Vp_N10_18_2154*	0.478	Hypothetical protein VIBHAR_00643
	*yejA*	*Vp_N10_18_2156*	0.494	Extracellular solute-binding protein
	*aapQ*	*Vp_N10_18_2529*	0.496	Amino acid ABC transporter permease
	*mlaF*	*Vp_N10_18_2720*	0.500	ATP-binding cassette domain-containing protein
	*proV*	*Vp_N10_18_0094*	2.147	Glycine betaine/L-proline transport ATP binding subunit
	*afuA*	*Vp_N10_18_1887*	2.243	Iron ABC transporter substrate-binding protein
	*fhuB*	*Vp_N10_18_1520*	2.270	Fe^3+^-hydroxamate ABC transporter permease FhuB
	*thiY*	*Vp_N10_18_1092*	2.400	Hypothetical protein
	*oppB*	*Vp_N10_18_3430*	2.402	Oligopeptide ABC transporter permease OppB
	*znuA*	*Vp_N10_18_4099*	2.594	Metal ABC transporter substrate-binding protein
	*artI*	*Vp_N10_18_0733*	3.101	Arginine ABC transporter substrate-binding protein
	*artM*	*Vp_N10_18_0735*	3.204	Arginine transporter permease subunit ArtM
	*thiZ*	*Vp_N10_18_1090*	3.271	Hydrogenase expression protein
	*thiX*	*Vp_N10_18_1091*	3.567	ABC transporter permease
	*-*	*Vp_N10_18_1522*	3.891	Iron (III) ABC transporter ATP-binding protein
	*artP*	*Vp_N10_18_0732*	4.015	Arginine ABC transporter ATP-binding protein ArtP
	*znuB*	*Vp_N10_18_1681*	6.403	Zinc ABC transporter permease subunit ZnuB
	*znuC*	*Vp_N10_18_1680*	9.190	Zinc ABC transporter ATP-binding protein ZnuC
	*znuA*	*Vp_N10_18_1679*	11.609	Zinc ABC transporter substrate-binding protein ZnuA
Propanoate metabolism	*puuE*	*Vp_N10_18_2902*	0.069	Aspartate aminotransferase family protein
	*prpE*	*Vp_N10_18_0011*	0.330	AMP-binding protein
	*acnD*	*Vp_N10_18_0013*	0.370	Fe/S-dependent 2-methylisocitrate dehydratase AcnD
	*pdhB*	*Vp_N10_18_0742*	0.382	Alpha-ketoacid dehydrogenase subunit beta
	*prpF*	*Vp_N10_18_0012*	0.431	2-Methylaconitate cis-trans isomerase PrpF
	*gabT*	*Vp_N10_18_0139*	0.432	4-Aminobutyrate--2-oxoglutarate transaminase
	*prpC*	*Vp_N10_18_0015*	0.438	2-Methylcitrate synthase
Benzoate degradation	*pcaH*	*Vp_N10_18_0737*	0.49	Dioxygenase family protein
	*pcaC*	*Vp_N10_18_2971*	2.003	Carboxymuconolactone decarboxylase family protein
Thiamine metabolism	*thiC*	*Vp_N10_18_4412*	2.116	Phosphomethylpyrimidine synthase ThiC
	*thiE*	*Vp_N10_18_4413*	2.247	Thiamine phosphate synthase
	*thiD*	*Vp_N10_18_1089*	2.555	Bifunctional hydroxymethylpyrimidine Kinase/phosphomethylpyrimidine kinase
	*tenA*	*Vp_N10_18_1094*	2.615	Thiaminase II
	*thiE*	*Vp_N10_18_1096*	2.740	Thiamine phosphate synthase
Fat digestion and absorption	*atoB*	*Vp_N10_18_3849*	0.371	3-Ketoacyl-CoA thiolase @ Acetyl-CoA Acetyltransferase
	*atoB*	*Vp_N10_18_2988*	4.215	Thiolase family protein
Quorum sensing	*-*	*Vp_N10_18_2155*	0.424	ABC transporter ATP-binding protein
	*ribA*	*Vp_N10_18_1217*	0.453	GTP cyclohydrolase II
	*-*	*Vp_N10_18_1879*	0.472	ABC transporter ATP-binding protein
	*-*	*Vp_N10_18_2632*	0.486	Sigma 54-interacting transcriptional regulator
	*-*	*Vp_N10_18_1219*	2.140	Sugar ABC transporter ATP-binding protein
	*-*	*Vp_N10_18_0181*	2.378	Polyamine ABC transporter substrate-binding protein
	*ribA*	*Vp_N10_18_2468*	2.675	GTP cyclohydrolase II
	*-*	*Vp_N10_18_1876*	2.918	ABC transporter permease
	*-*	*Vp_N10_18_2783*	9.727	ABC transporter permease
Pathogenic *Escherichia coli* infection	*gapA*	*Vp_N10_18_3876*	2.086	Glyceraldehyde-3-phosphate dehydrogenase
	*yscF*	*Vp_N10_18_0060*	5.836	Type III secretion system needle filament protein VscF

## Data Availability

The draft genome of *V. parahaemolyticus* N10-18 is available in the GenBank database under the accession number JALGSE000000000. A complete list of DEGs in the strain under the Cd^2+^ stress is available in the National Center for Biotechnology Information (NCBI, https://www.ncbi.nlm.nih.gov, accessed on 21 April 2022) SRA database under the accession number PRJNA825334. Other data is contained within the article or Supplementary Materials.

## Supplementary Materials

The following supporting information can be downloaded at: https://www.mdpi.com/article/10.3390/foods11233777/s1, Figure S1: The k-mer analysis for *V. parahaemolyticus* N10-18 subread data based on the number of unique 17-mers; Table S1: The genotype and phenotype of *V. parahaemolyticus* N10-18 isolate used in this study; Table S2: The 65 *V. parahaemolyticus* strains analyzed in the phylogenetic tree; Table S3: The identified GIs, Ins, and ISs in the *V. parahaemolyticus* N10-18 genome; Table S4: The identified repeats in the *V. parahaemolyticus* N10-18 genome [104,105,106,107]; Table S5: The potential virulence-associated genes identified in the *V. parahaemolyticus* N10-18 genome; Table S6: Oligonucleotide primers used in the RT-PCR assay; Table S7: Expression of representative DEGs by the RT-PCR assay.
