# Supplementary material for: Genomic and Transcriptomic Analysis Reveal Multiple Strategies for the Cadmium Tolerance in Vibrio parahaemolyticus N10-18 Isolated from Aquatic Animal Ostrea gigas Thunberg
Source: Foods. 2022 Nov 23;11(23):3777. doi: 10.3390/foods11233777 (PMC9741282; doi:10.3390/foods11233777)
Supplement: Supplementary file 1 [file foods-11-03777-s001.zip › foods-1948570-supplementary.pdf]

Supplementary materials

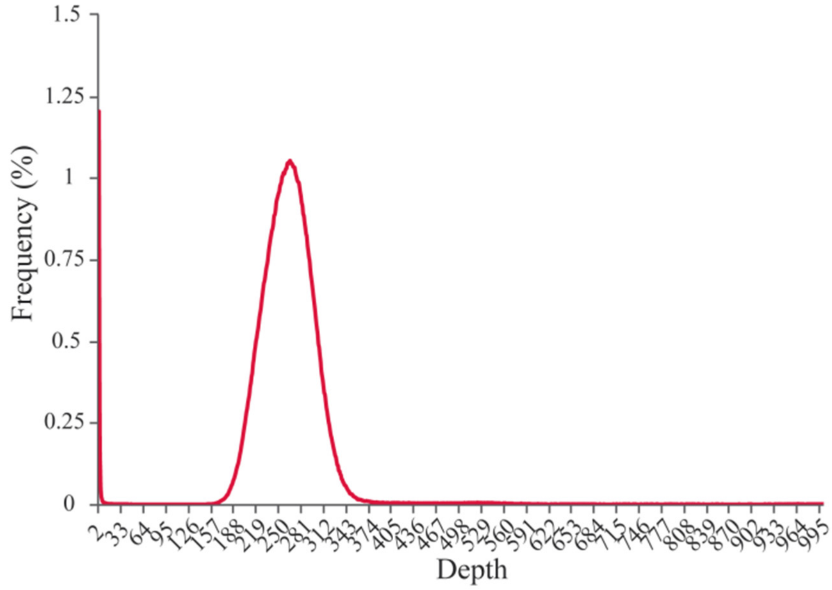

Figure S1. The k-mer analysis for *V. parahaemolyticus* N10-18 subread data based on the number of unique 17-mers.

Table S1. The genotype and phenotype of *V. parahaemolyticus* N10-18 isolate used in this study.

Feature	***V. parahaemolyticus* N10-18**
Source	*Ostrea gigas thunberg*
Genotype	*tdh*⁻/*trh*⁻
Heavy metal resistance phenotype	Cd^{2+}/Zn^{2+}
Antibiotic resistance phenotype	AMP/KAN//STR
MIC value (μg/mL)	
Cd^{2+}	400
Zn^{2+}	1,600
AMP	50,000
KAN	128
STR	128

Table S2. The 65 *V. parahaemolyticus* genomes analyzed in the phylogenetic tree.

V. parahaemolyticus Strain	Source	Collected Date	Serotype	ST	Location	GenBank accession no.
64	*Penaeus vannamei*	2018	O1: KUT	1	China	GCA_019321785.1
10329	Homo sapiens	Missing	O4/O12:K12	36	Missing	GCA_009649015.1
160807	*Penaeus vannamei*	2016	O1: KUT	809	China	GCA_003691525.1
19-021-D1	*Penaeus vannamei*	2019	O1: KUT	413	SouthKorea	GCA_009734325.1
20_082A3	*Penaeus vannamei*	2020	O1: KUT	413	SouthKorea	GCA_015779285.1
20-082E4	*Penaeus vannamei*	2021	O1: KUT	413	SouthKorea	GCA_020041945.1
2010V-1106	Homo sapiens	2010	O4/O12:K12	36	Missing	GCA_009764075.1
2012AW-0154	Missing	Missing	O3: KUT	1727	USA	GCA_009665495.1
2012AW-0224	Missing	2012	O2: KUT	679	USA	GCA_009764055.1
2012AW-0353	Missing	2012	O3: KUT	1727	USA	GCA_009763445.1
20130629002S01	*Penaeus vannamei*	2013	O1: KUT	1166	China	GCA_003085735.1
2013V-1136	Missing	Missing	O1: KUT	NAN	Missing	GCA_009763605.1
2013V-1146	Homo sapiens	2013	O4/O12:K12	36	USA	GCA_009763645.1
2013V-1174	Homo sapiens	2013	O4/O12:K55	34	USA	GCA_009763625.1
2013V-1181	Homo sapiens	2013	O11: KUT	631	USA	GCA_009763565.1
2013V-1244	Homo sapiens	2013	O1:K33/K55	162	USA	GCA_009763545.1
20140624012-1	*Penaeus vannamei*	2014	O3:KUT	NAN	China	GCA_009883815.1
20140722001-1	*Penaeus japonicus*	2014	O3:KUT	415	China	GCA_009883835.1
20140829008-1	*Penaeus vannamei*	2014	O1:KUT	415	China	GCA_009883855.1
2014V-1066	Homo sapiens	2014	O4/O12:K12	36	USA	GCA_009763525.1
2014V-1125	Homo sapiens	2014	O4/O12:K12	36	USA	GCA_009763505.1
20151116002-3	*Penaeus japonicus*	2015	O1:KUT	1166	China	GCA_009883895.1
2015AW-0174	Homo sapiens	2015	O4/O12:K12	36	USA	GCA_009763165.1
20160303005-1	*Penaeus vannamei*	2016	O1:K68	NAN	China	GCA_009883875.1
AM43962	Missing	Missing	O10/O12:K41	NAN	USA	GCA_009763025.1
AM46865	Homo sapiens	Missing	O8:K41	1815	USA	GCA_009763425.1
AM51552	Missing	Missing	O4/O12:K34	NAN	USA	GCA_009763405.1
ATCC_17802	Environment	1951	O1:K1	1	Japan	GCA_001558495.2
BB22OP	Environment	1980	O4:K8	88	Bangladesh	GCA_000328405.1
BTXS2	Environment	2019	O5:K30	NAN	China	GCA_015172915.1
CDC_K4557	Homo sapiens	2006	O1:K33/K55	799	USA	GCA_000430425.1
CHN25	Shrimps	2011	O5:K17	395	China	GCA_001700835.1
D3112	Environment	2015	OUT: K32	NAN	China	GCA_004194515.1
FB-11	*Penaeus vannamei*	2017	O1: KUT	165	China	GCA_018135645.1
FDA_R31	Oyster	2007	O1: KUT	23	USA	GCA_000430405.1
FDAARGOS_191	Homo sapiens	1996	O3:K6	3	India	GCA_002073775.2
FDAARGOS_51	Homo sapiens	1998	O4/O12:K12	36	USA	GCA_001188185.2
FDAARGOS_662	Homo sapiens	Missing	O4/O12:K12	36	USA	GCA_008693745.1
FDAARGOS_667	Homo sapiens	Missing	O5:K33/K55	NAN	USA	GCA_008693625.1
FORC_004	Environment	2014	O6: KUT	1628	SouthKorea	GCA_001433415.1
FORC_006	Missing	2014	O2: KUT	1630	SouthKorea	GCA_001304775.1
FORC_008	finespotted flounder	2014	O8:K41	984	SouthKorea	GCA_001244315.1
FORC_014	Toothfish	2015	O5: KUT	1629	SouthKorea	GCA_001636035.1
FORC_018	Sea-bass	Missing	O8:K41	984	SouthKorea	GCA_001887055.1
FORC_022	Marinated Crab	Missing	O3:KUT	233	SouthKorea	GCA_001879585.1
FORC_023	Homo sapiens	2014	O5:KUT	2015	SouthKorea	GCA_001758605.1
FORC_071	Missing	2017	O11:KUT	NAN	SouthKorea	GCA_003612715.1

FORC_072	Missing	2017	O2:K28	NAN	SouthKorea	GCA_003612695.1
LVP_1	Crayfish	2013	O2:KUT	2150	China	GCA_013393845.1
LVP_2	Crayfish	2013	O1:KUT	NAN	China	GCA_013393865.1
LVP_66	Crayfish	2013	O1:KUT	1798	China	GCA_013393885.1
MAVPQ	Homo sapiens	2011	O11:KUT	631	China	GCA_001682175.1
MAVP_Q	Homo sapiens	2011	O11:KUT	631	Missing	GCA_002209725.2
MAVP_R	Homo sapiens	2011	O11:K15	NAN	Missing	GCA_002220985.3
MVP_1	Environment	2016	O1:K68	1913	Malaysia	GCA_001996365.2
N10-18	*Ostrea gigas thun-berg*	2017	O4/O11:K4	499	China	This_Study
PB1937	Shrimp	2012	O1:KUT	150	China	GCA_003351885.1
R13	*Penaeus vannamei*	2016	O6:KUT	NAN	PacificOcean	GCA_003119375.1
R14	*Penaeus vannamei*	2016	O6:KUT	NAN	PacificOcean	GCA_003076895.1
RIMD_2210633	Homo sapiens	1996	O3:K6	3	Japan	GCA_000196095.1
S107-1	Missing	Missing	O3:K12	NAN	Canada	GCA_003047085.1
TJA114	*Penaeus vannamei*	2017	O8:KUT	1743	China	GCA_014217295.1
UCM-V493	Environment	2002	O2:K28	471	Spain	GCA_000568495.1
Vb0624	Missing	2015	O7/O12:K6	163	China	GCA_006517795.1
VP_D14	Missing	2012	O3:K6	3	China	GCA_004006515.1

Table S3. The identified GIs, INs and ISs in the *V. parahaemolyticus* N10-18 genome.

MGE	MGE ID	Location	Scaffold Size (bp)	Start (bp)	End (bp)	CDS Gene
GI	GI 1	Scaffold 11	174,719	2,878	18,078	15
	GI 2	Scaffold 14	108,661	88,409	103,363	13
IN	IN 1	Scaffold 6	241,503	238,264	240,830	2
	IN 2	Scaffold 7	227,777	165	227,764	210
	IN 3	Scaffold 28	16,301	393	16,270	24
	IN 4	Scaffold 35	5,031	118	4,013	5
	IN 5	Scaffold 36	4,792	395	4,779	6
	IN 6	Scaffold 39	4,094	109	3,975	5
	IN 7	Scaffold 42	2,491	600	1,900	2
	IN 8	Scaffold 45	1,727	360	1,270	2
IS	IS001	Scaffold 29	12,178	1,418	2,745	1

Table S4. The potential virulence-associated genes identified in the *V. parahaemolyticus* N10-18 genome.

Gene	Description	Reference
kdsA	2-dehydro-3-deoxyphosphooctonate aldolase	[64]
gmd	GDP-mannose 4,6-dehydratase	[63]
ilpA	immunogenic lipoprotein A	[60]
MAM7	multivalent adhesion molecule 7	[61]
gmhA	phosphoheptose isomerase	[62]
tlh	thermolabile hemolysin TLH	[49]
exsA	transcriptional regulator ExsA	[86]
exsC	transcriptional regulator ExsC	[86]
exsD	transcriptional regulator ExsD	[86]
vscN	type III secretion system ATPase VscN	[86]
vcrD	type III secretion system C ring protein VcrD	[104]
vscT	type III secretion system C ring protein VscT	[104]
vcrG	type III secretion system chaperone VcrG	[104]
vcrH	type III secretion system chaperone VcrH	[104]
vecA	Type III secretion system chaperone VecA	[105]
vscG	Type III secretion system chaperone VscG	[104]
vscW	Type III secretion system chaperone VscW	[105]
vscR	type III secretion system C-ring protein VscR	[104]
vscS	type III secretion system C-ring protein VscS	[104]
vscU	type III secretion system C-ring protein VscU	[104]
vscX	type III secretion system C-ring protein VscX for secretion specificity	[104]
vscY	type III secretion system C-ring protein VscY for secretion specificity	[104]
vscK	type III secretion system cytoplasmic protein VscK	[104]
vscL	type III secretion system cytoplasmic protein VscL	[104]
vscQ	type III secretion system cytoplasmic protein VscQ	[104]
vopQ	type III secretion system effector VopQ	[104]
/	VPA0450 family T3SS effector inositol phosphatase	
vopR	Type III secretion system effector VopR	[104]
vopS	type III secretion system effector VopS	[104]
vscD	type III secretion system IM ring protein VscD	[104]
vscJ	type III secretion system IM ring protein VscJ	[104]
vscI	type III secretion system inner rod protein VscI	[104]
vscP	type III secretion system needle length control protein VscP	[104]
vscF	type III secretion system needle protein VscF	[104]
vcrV	type III secretion system needle tip protein VcrV	[104]
vscC	type III secretion system OM ring protein VscC	[104]
vcrR	type III secretion system protein VcrR	[104]
vopN	type III secretion system protein VopN	[106]
vscH	type III secretion system protein VscH	[104]
yscO	type III secretion system protein YscO	[107]
tyeA	TyeA family type III secretion system gatekeeper subunit	[104]
sycN	type III secretion chaperone SycN	[104]
vscB	YscB family type III secretion system chaperone VscB	
vopB	type III secretion system translocator protein VopB	[104]
vopD	type III secretion system translocator protein VopD	[104]

Table S5. The identified repeats in the *V. parahaemolyticus* N10-18 genome.

Repeat ID	Location	Scaffold Size (bp)	Repeat				
			Start	End	Length (bp)	Size (bp)	Copy No.
**Scaffold1_TR01**	**Scaffold 1**	587,332	29,533	29,578	46	21	2.2
Scaffold2_TR03	Scaffold 2	573,558	571,252	571,302	51	6	8.5
Scaffold3_TR05	Scaffold 3	557,129	447,180	447,219	40	6	6.7
Scaffold4_TR11	Scaffold 4	384,640	367,028	367,091	64	7	9.1
Scaffold5_TR12	Scaffold 5	360,589	347,209	347,720	512	256	2
Scaffold6_TR14	Scaffold 6	241,503	237,484	237,527	44	9	4.9
Scaffold9_TR16	Scaffold 9	194,495	128,107	128,790	684	342	2
Scaffold10_TR17	Scaffold 10	177,419	129,106	129,177	72	24	3
Scaffold11_TR23	Scaffold 11	174,719	123,775	124,090	316	115	2.7
Scaffold12_TR26	Scaffold 12	162,127	149,472	149,528	57	24	2.4
Scaffold13_TR29	Scaffold 13	122,373	72,353	72,934	582	251	2.3
Scaffold14_TR30	Scaffold 14	108,661	91,020	91,090	71	33	2.2
Scaffold18_TR31	Scaffold 18	75,503	67,524	67,784	261	131	2
Scaffold20_TR32	Scaffold 20	60,684	370	827	458	229	2
Scaffold24_TR33	Scaffold 24	34,923	17,263	17,310	48	6	8
Scaffold25_TR34	Scaffold 25	31,664	4,529	4,583	55	6	9.2
Scaffold26_TR35	Scaffold 26	24,272	20,208	20,599	392	212	1.8
Scaffold28_TR36	Scaffold 28	16,301	13,702	13,867	166	82	2
Scaffold29_TR37	Scaffold 29	12,178	9,940	10,329	390	195	2
Scaffold31_TR39	Scaffold 31	11,167	10,108	11,167	1,060	366	2.9
Scaffold39_TR40	Scaffold 39	4,094	2,963	3,271	309	155	2
Scaffold41_TR41	Scaffold 41	3,344	2,095	2,237	143	72	2
Scaffold42_TR42	Scaffold 42	2,491	1,586	1,797	212	106	2

Table S6. Oligonucleotide primers used in the RT-PCR assay.

Target gene	Primer	Sequence (5′ to 3′)	Predicted size (bp)	Reference
znuA	*Vp_N10_18_1679*-F	TTGCGGCTAAAGATAAGGAA	138	In this study
	Vp_N10_18_1679-R	CGGGAGTCACCGTAAAGTGT		
znuC	*Vp_N10_18_1680*-F	CTGCTACAACGCCCTGACTT	208	In this study
	Vp_N10_18_1680-R	GTGATCCCGAACAACAAACG		
tenA	*Vp_N10_18_1694*-F	GCCCACCATGTTACCTACTGC	277	In this study
	Vp_N10_18_1094-R	CCACGCCTGATTGGAACTCTT		
16s RNA	*16s RNA*-F	GACACGGTCCAGACTCCTAC	179	[25]
	16s RNA-R	GGTGCTTCTTCTGTCGCTAAC		

Table S7. Expression of representative DEGs by the RT-PCR assay.

Gene	Predicted protein	Fold change	
		RNA-Seq.	RT-PCR
Vp_N10_18_1679	Zinc ABC transporter substrate-binding protein ZnuA	11.609	20.956
Vp_N10_18_1680	Zinc ABC transporter ATP-binding protein ZnuC	9.190	9.626
Vp_N10_18_1094	Thiaminase II	2.615	2.043
